# Effects of jump training on power, strength, balance and aerobic performance in non-exercising young adults

**DOI:** 10.3389/fspor.2026.1746624

**Published:** 2026-02-26

**Authors:** Markus Gruber, Justin Howaldt, Michael Schwenk, Lorenz Assländer, Maria Moreno-Villanueva, Maik Bieleke, Julia Schüler, Luca Ruggiero, Philipp Barzyk

**Affiliations:** Department of Sport Science, University of Konstanz, Konstanz, Germany

**Keywords:** cardiorespiratory, countermeasure, exercise, motor learning, neuromuscular, countermovement jump

## Abstract

Physical inactivity is a major risk factor for noncommunicable diseases and associated mortality. A high-intensity jump training has proven to be efficient in counteracting inactivity related declines in physical function during two months of bed rest. In the present study, we tested the effects of such a training program under real-life conditions. Seventy-five young adults (38 female, 23 ± 3 years) were randomly assigned to either a training or a control group. The training group underwent an 8-week jump training with 15 min of exercises, 3 days per week. Before and after the 8-week period as well as another 8 weeks later we tested jump performance in countermovement jumps (CMJ) and hops, balance performance, maximal isometric strength of leg extensor muscles, stair climb performance, gait analysis, and peak oxygen uptake in a cardiopulmonary exercise test. We observed training specific increases (8% ± 9%) in CMJ height and peak power (5% ± 7%) that were explained by an optimized movement technique. We did not observe generic improvements of balance, strength or functional performance after the training. Peak oxygen uptake showed increases for participants exhibiting low baseline levels. A low-volume high-intensity jump training program was sufficient to increase neuromuscular power and performance in the trained task. Improvements of peak oxygen uptake were restricted to participants with low aerobic capacity baseline levels. These findings suggest that such a program has the potential to induce more generic adaptations and improve the anaerobic and aerobic power of previously non-exercising individuals through a baseline-dependent tailoring of training volume.

## 1 Introduction

There is clear evidence that physical activity comes along with huge benefits on health and longevity (1, 2). Notwithstanding such evidence and decades of tremendous political endeavors to increase physical activity in the population, a large and still increasing proportion of people is insufficiently active (3). A recent meta-analysis on studies using wearable devices indicated a downward trend in physical activity in the order of −600 steps per day per decade for adults and −1,500 steps per day per decade for adolescents since 1995 (4). The consequences are disastrous: physical inactivity and subsequent declines in physical function will continue to increase in significance as a major risk factor for non-communicable diseases (5), leading to an ever-increasing economic burden (6) and mortality rate (7).

A seamless alternative to programs that aim at increasing physical activity are exercise regimes which have the inherent potential to counteract the negative consequences of physical inactivity. The best available experimental model to study exercise countermeasures against detrimental effects of physical inactivity in a healthy population is bed rest (8, 9). Within the last few decades, a large number of potential exercise programs to mitigate the effects of bed rest on the cardiovascular, the neuromuscular and the skeletal system, have been tested. However, most of the tested countermeasures were specifically targeting only one of those physiological systems (10). Recently we developed and implemented a jump exercise countermeasure during a 60-day bed rest, designed to address all physiological systems in an integrative exercise approach (11).

The follow-up to this study has clearly shown that such a training significantly mitigated the negative effects of bed rest on the cardiovascular system (12–14) and was even more effective than other exercise approaches in preserving bone and muscle mass (11), which may be mediated by reported endocrine responses to jump training (15), muscle structure, phenotype and myofiber oxidative capacity (16) as well as muscle power (17) and strength (13). In addition, the jump training demonstrated its potential as an integrated countermeasure as it prevented declines in postural control, gait characteristics and functional mobility (18). Therefore, with regards to the generic and significant effects, despite the short duration of only 15 min of training for 5 days a week, high impact repetitive exercises can be considered most efficient in counteracting inactivity related degradations in physical performance (9).

While bed rest provides a controlled model of complete physical inactivity, translating effective countermeasures from the confines of the laboratory to the general population is a critical next step. In the present study, the training program was transferred to a real-life scenario. We tested the effects of a repetitive jump training program on healthy non-exercising young adults under habitual living conditions. With only 3 training sessions of 15 min per week, but at high to maximal intensity, we tried to minimize volume, while providing a sufficient stimulus for training adaptations. As our primary outcome, we hypothesized to find increases in jump performance in the training group vs. our control group. As secondary outcomes, we looked at effects on strength, balance, functional motor performance, and aerobic capacity to assess the program's potential to improve overall physical performance.

## 2 Materials and methods

### 2.1 Participants

**Figure 1:**
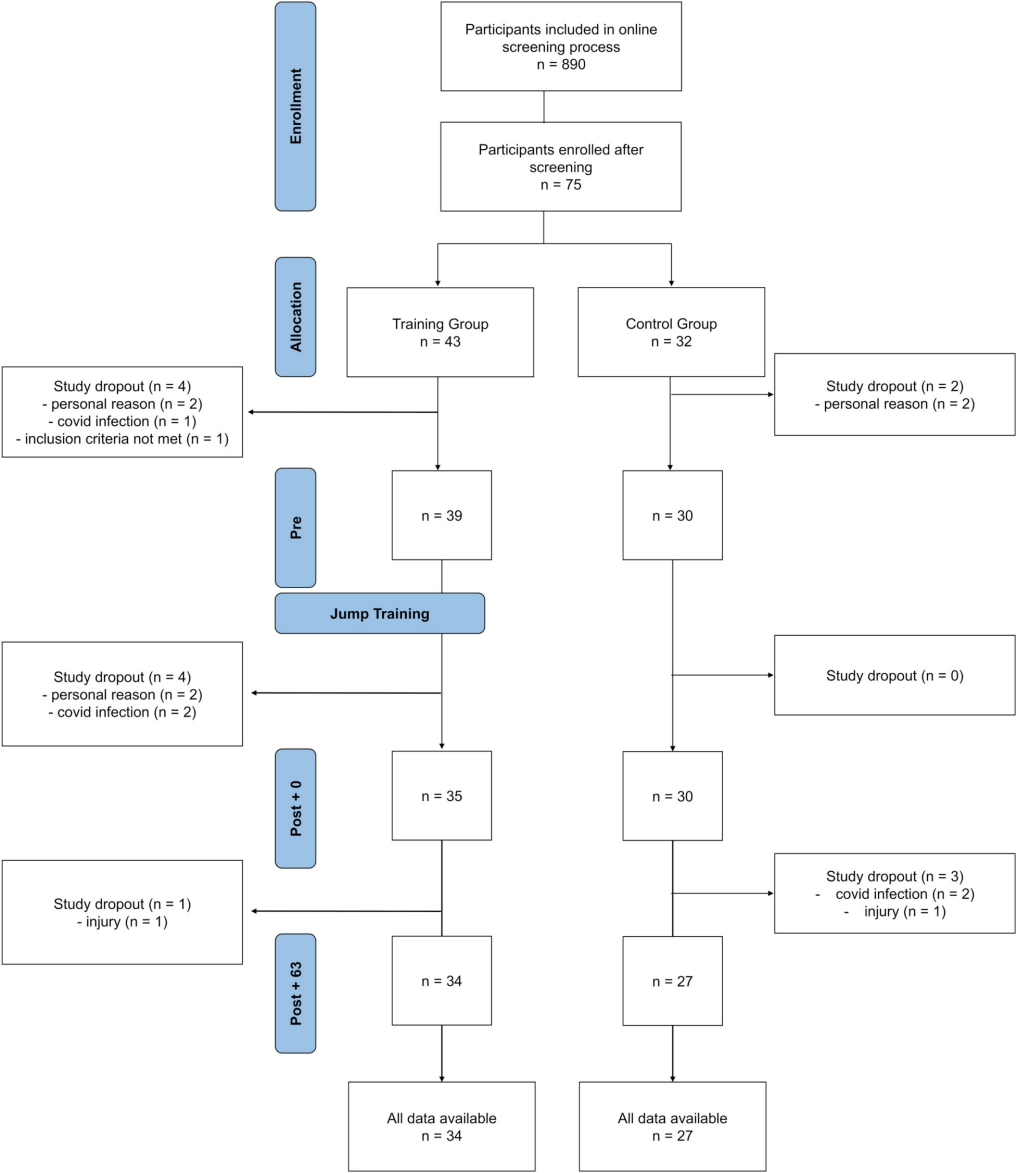
The flowchart illustrates the enrollment, allocation, and follow-up process of participants in the study. Physical performance was tested before the training (Pre), directly after the 8-week training program (Post + 0) and another 8 weeks later (Post + 63). Please note that dropouts occurred at various stages due to different reasons, which were all unrelated to the study itself.

The study focused on adults who were not involved in an exercise program at the time of recruitment. Participants were selected based on continuous review of screening questionnaires, and individuals with the lowest relative scores on validated measures of physical activity and fitness, including the stage of change in physical activity (19), weekly moderate-to-vigorous physical activity (MVPA) according to the short version of the International Physical Activity Questionnaire (IPAQ) (20), self-rated physical fitness (21), and self-rated fitness percentile, were preferentially invited. Further exclusion criteria were any acute injuries, physical illnesses, mental illnesses, medication use, and any other medical conditions that did not allow jump exercises. The training study was part of a larger study in which the participants carried out further experiments for which additional exclusion criteria were set (e.g., no tattoos due to MRI requirements). Out of the 890 individuals who completed an online screening, 75 met inclusion criteria and were enrolled in the study. Sixty-five of those enrolled completed the Post + 0 measurements and were therefore included in further analysis (see Figure 1 for details). These 65 participants reported a stage of change in physical activity of 2.6 ± 0.7 (Likert scale from 1 = precontemplation to 5 = maintenance), MVPA of 137 ± 197 min/week, a self-rated physical fitness level of 2.6 ± 0.7 (Likert scale from 1 = very poor to 5 = very good), and a self-rated physical fitness percentile of 32 ± 16 during screening.

The study was approved by the Ethics Committee of the University of Konstanz (No. 31/2022) and conducted in accordance with the latest revision of the Declaration of Helsinki. At Pre, the 65 participants (34 females, age 23 ± 3 years, body height 173 ± 8 cm, body mass 71 ± 12 kg, BMI 23.7 ± 3.1, resting heart rate 78 ± 13 min^−1^, systolic blood pressure 125 ± 13 mmHg, diastolic blood pressure 83 ± 10 mmHg) signed an informed consent form, after we provided detailed verbal and written information about the study design and procedures, exclusion criteria and potential risks.

We randomly assigned participants to a training group (*n* = 35; 18 females, age 23 ± 3 years, body height 172 ± 8 cm, body mass 70 ± 10 kg, BMI 23.4 ± 3, resting heart rate 78 ± 13 min^−1^, systolic blood pressure 125 ± 15 mmHg, diastolic blood pressure 84 ± 11 mmHg) or a control group (*n* = 30; 16 females, age 23 ± 2 years, body height 173 ± 9 cm, body mass 73 ± 14 kg, BMI 24.1 ± 3.4, resting heart rate 78 ± 13 min^−1^, systolic blood pressure 124 ± 11 mmHg, diastolic blood pressure 82 ± 9 mmHg) using a computer-generated randomization sequence. Participants of the training group had reported a stage of change in physical activity of 2.7 ± 0.7, MVPA of 140 ± 170 min/week, a self-rated physical fitness level of 2.7 ± 0.7, and a self-rated physical fitness percentile of 35 ± 15 during screening. Corresponding values in the control group were 2.6 ± 0.6, 134 ± 228 min/week, 2.6 ± 0.7, and 30 ± 17, respectively. As we assumed a higher drop-out rate in the training group, we assigned 30 per cent more participants to this group. The participants in the training group performed high-intensity jump training 3 times a week for 8 weeks. At Pre, we administered a battery of tests to assess the physical baseline performance of all participants. After 8 weeks (Post + 0; 0 days after the intervention) and 16 weeks (Post + 63; 63 days after the intervention), we reassessed all participants using the same set of tests with the identical order as in the Pre session.

### 2.2 Performance tests

We measured body mass and height at the beginning of each test session using a calibrated mechanical seca 700 scale with an integrated vertical measuring rod and headpiece (seca, Hamburg, Germany). Afterwards, participants performed a warm-up consisting of 30 s of jumping jacks, 30 s of countermovement jumps (CMJs), and 30 s of high knee running with 30 s of rest between tasks. All exercises were initially demonstrated by the experimenter.

After the warm-up, we assessed CMJ performance, hop performance, and static balance of participants using a Leonardo Mechanograph® GRFP force plate (Novotec Medical GmbH, Pforzheim, Germany) operating at 800 Hz. First, participants performed 3 maximal CMJs with their hands on their hips. We instructed them to stand still until they heard a loud beep, after the beep, they should perform the CMJ with the aim of jumping as high as possible while keeping the legs straight during the flight phase. Participants had a 1-min break between each jump. We used custom written MATLAB© scripts (MathWorks, Natick, MA, USA) to analyze all force traces off-line and conducted all analyses according to previous methodology (22); for details see also Supplementary Material S1. For in depth analysis of jump kinetics we chose specifically the following variables: jump height [m], absolute [kW] and relative (to body mass) [W kg^−1^] peak power, absolute [kN] and relative [N kg^−1^] force at zero velocity, upward movement range [m], and upward and braking movement duration [ms]. Jump height was calculated using the impulse-momentum method. For comparability with previous literature using field-based methods, jump height was also calculated using flight time (23) (Supplementary Material S1). For statistical comparisons, we calculated the average of the 3 CMJs for each variable.

Following the CMJs, participants performed 2 sets of 10 reactive hops with the instruction to hop as high as possible and to keep ground contact time as short as possible. From each hop trial, hops 4–8 were retained for further analysis. The following variables were calculated, and averaged across the 5 selected hops, and the two trials: contact and flight time [ms], reactive strength index (ratio of flight to contact time), hop height [m], absolute [kN] and relative [N kg^−1^] peak force. The calculations were performed using previously reported methods and algorithms (11) (Supplementary Material S2).

Thereafter, participants performed a single-leg standing test to assess static balance. We instructed participants to stand as still as possible on their dominant leg (determined as the leg used to kick a ball) and keep their hands on their hips for 15 s. We measured the change in position of the center of pressure (CoP) on the force plate and derived an ellipse with the area covering 95% of the CoP path [cm^2^].

We used a dynamometer (Isomed 2000, D&R Ferstl GmbH, Nuremberg, Germany) to determine maximum isometric leg press strength. Participants were positioned in a leg press module with 60° hip flexion, 90° knee flexion and 90° ankle plantarflexion. The leg press was equipped with a force plate to measure ground reaction forces during the maximal leg extension trials. Participants were instructed to “push themselves off as hard as possible from the force plate”. Each trial lasted 3 s, and we repeated it 3 times, with 30 s rest between trials. The positioning data for each participant was stored in the dynamometer, but technical issues led to incorrect standardization for some participants. As a result, we needed to exclude leg press data from 14 training group participants at Pre and Post + 0, and 15 at Post + 63. In the control group, we needed to exclude 18 at Pre and Post + 0, and 17 at Post + 63. Thus, we ended up with 31 participants in total who were included in the final analysis of leg press data. We took the maximum force value from each trial and determined the MVC for each participant as the mean of the maximum force values of the 3 trials.

To assess potential transfer effects in activities of daily living, we conducted a 14-step stair climb and a gait test. The 14-step stair climb was performed at habitual speed. Prior to the stair climb, we asked participants to stand still for 2 min. Immediately, after the 2 min we measured their heart rate [min^−1^] by a sports watch (Polar Vantage 2©, Polar OY, Kempele, Finland) with a chest strap and a heart rate monitor (Polar M10 HR-monitor©, Polar OY, Kempele, Finland). Thereafter, the participants climbed up the stairs, and we assessed the climbing time [s] with a stopwatch. Then participants stood still, and we measured the heart rate peak [min^−1^] within a 30 s-window after the stair climb test. Following the stair climb test, we conducted 8 gait test trials using the OptoGait© system (Microgate©, Bolzano, Italy) over a 10 m corridor. We instructed participants to walk at an “everyday pace, as if walking calmly to the bus stop”. We extracted gait variables from the proprietary OptoGait© software, which provides the mean plus standard deviation for stride time [s], stride length [m] and mean walking speed [ms^−1^] of the 8 trials.

Lastly, we performed a cardiopulmonary exercise test (CPET). Participants laid down for 5 min and we determined resting heart rate [min^−1^] (Polar M10 HR-monitor©, Polar OY, Kempele, Finland) and blood pressure (Boso medicus X©, BOSCH + SOHN GmbH u. Co. KG, Jungingen, Germany). Thereafter, participants performed an incremental ramp test on a cycle-ergometer (Ergoline GmbH, Bitz, Germany) while we measured heart rate and breath-by-breath respiratory gas exchange with an Ergostik© gas analyzer (Geratherm Respiratory GmbH, Bad Kissingen, Germany) and the Blue Cherry© diagnostic platform. The test started with a 5-min warm-up at 35 W, during which we instructed participants to “ride straight ahead for 5 min on a flat road at a comfortable everyday pace as if taking a leisurely Sunday stroll”. After the 5min warm-up, power output increased by 3 W every 12 s (15 W per min) and participants kept their cadence between 70 and 80 min^−1^. We determined exhaustion when participants could no longer maintain 60 min^−1^, or until they could go no further. Further, we determined $\dot{V}O_{2\mathrm{peak}}$ [ml kg^−1^min^−1^] as the peak value of a 30-s moving average of breath-by-breath oxygen uptake. We determined peak power [W] and peak heart rate [min^−1^] as the maximum values that were achieved during the test. During off-line analysis we identified artifacts in O2 and CO2 breath-by-breath data that affected $\dot{V}O_{2\mathrm{peak}}$ detection, likely due to equipment failure or incorrect mask positioning. In total we needed to exclude five measurements, which led to a drop-out of five participants specifically for the CPET (3 control, 2 training).

### 2.3 Jump training protocol

The participants of the training group performed jump training sessions of 15 min on 3 days a week for 8 weeks. We instructed the participants of the control group to maintain their normal daily routine for 8 weeks and they did not receive any training or any other intervention during this time. For the training group, we gradually increased the intensity and volume of the training, with a minimum of 24 h rest between 2 sessions. We instructed the training group to perform 3 sessions per week, with 1 session taking place under controlled laboratory conditions at the Human Performance Research Centre (HPRC), University of Konstanz. We fully instructed the participants on how to perform the remaining 2 weekly sessions, which were performed at home. Each training session consisted of 4 parts: a standardized warm-up (30 s jumping jacks, 30 s submaximal CMJs, and 30 s high knee running), hops, CMJs and high-intensity CMJ training. We varied CMJs and hops in number of repetitions and length of rest to progressively increase the training effort throughout the training program (see Supplementary Material S3 for information about the training programming per session). We thoroughly explained all parts of the training during the first training session, which was held at the HPRC. We further provided a detailed training plan to the participants (Supplementary Material S4) containing video links that allowed participants to review the warm-up and exercises at home.

We provided all participants with a sports watch (Polar Vantage 2©, Polar OY, Kempele, Finland) to store and track their training units. The watch guided the participants through each training session, automatically indicating the start and end of each exercise, as well as the duration, number of repetitions and length of breaks. Jump training adherence was defined as the number of recorded jump training sessions lasting longer than 10 min, expressed relative to the prescribed total of 24 sessions. Peak training intensity was calculated as the peak heart rate during each session, expressed as a percentage of the individual's peak heart rate determined during the CPET at Pre. While this measure reflects cardiovascular training intensity, neuromuscular load was maximized by instructing participants to perform all jumps with maximum effort.

### 2.4 Statistical analysis

We conducted all statistical analyses in R (version R4.2.2) and created figures using ggplot2 or MATLAB© (TheMathworks, Natick, USA). To test for baseline differences between groups, we ran independent *t*-tests for all variables at Pre using the R stats package. To examine the effects of training over time, we computed linear mixed effects models using the lmer function (lme4 package). We treated performance measures as dependent variables, with fixed effects for time (Pre, Post + 0, Post + 63), group (training, control), and their interaction (time × group). We added a random intercept for participants to account for within-subject variability and evaluated model significance by type III ANOVA using the Anova function (car package). In case of significant group × time interaction effects, we executed *post-hoc* pairwise comparisons with estimated marginal means (emmeans) using the Bonferroni correction. We calculated effect sizes as Cohen's *d* for pairwise comparisons and partial eta squared (*η*^2^) for ANOVA main and interaction effects.

To gain more detailed insights into the observed changes in CMJ technique, we applied functional principal component analysis (24) to the normalized relative force-time traces, which is an unsupervised learning technique used to identify the functions explaining the greatest variance in relative force from pre-training to Post + 0. Details on this procedure are provided in Supplementary Material S5.

To explore relationships between baseline scores and changes in CMJ and aerobic performance, we calculated pre-post delta scores for CMJ height and $\dot{V}O_{2\mathrm{peak}}$, e.g., $\dot{V}O_{2\mathrm{peak}}$ changes as (($\dot{V}O_{2\mathrm{peak}}$ post − $\dot{V}O_{2\mathrm{peak}}$ pre)/($\dot{V}O_{2\mathrm{peak}}$ pre × 100)). We used Pearson's correlation coefficients for analysis and visualized the relationships with scatter plots. Furthermore, we fitted linear regression models to the delta plots and plotted confidence intervals using geom_smooth in ggplot2.

## 3 Results

The groups did not differ significantly on any of the measured variables in the pre-test (Supplementary Material S6). Participants of the training group recorded an average of 22 ± 4 jump training sessions, corresponding to a mean adherence rate of 92% ± 17%. The relative peak intensity across all training sessions was 92% ± 5%.

**Figure 2:**
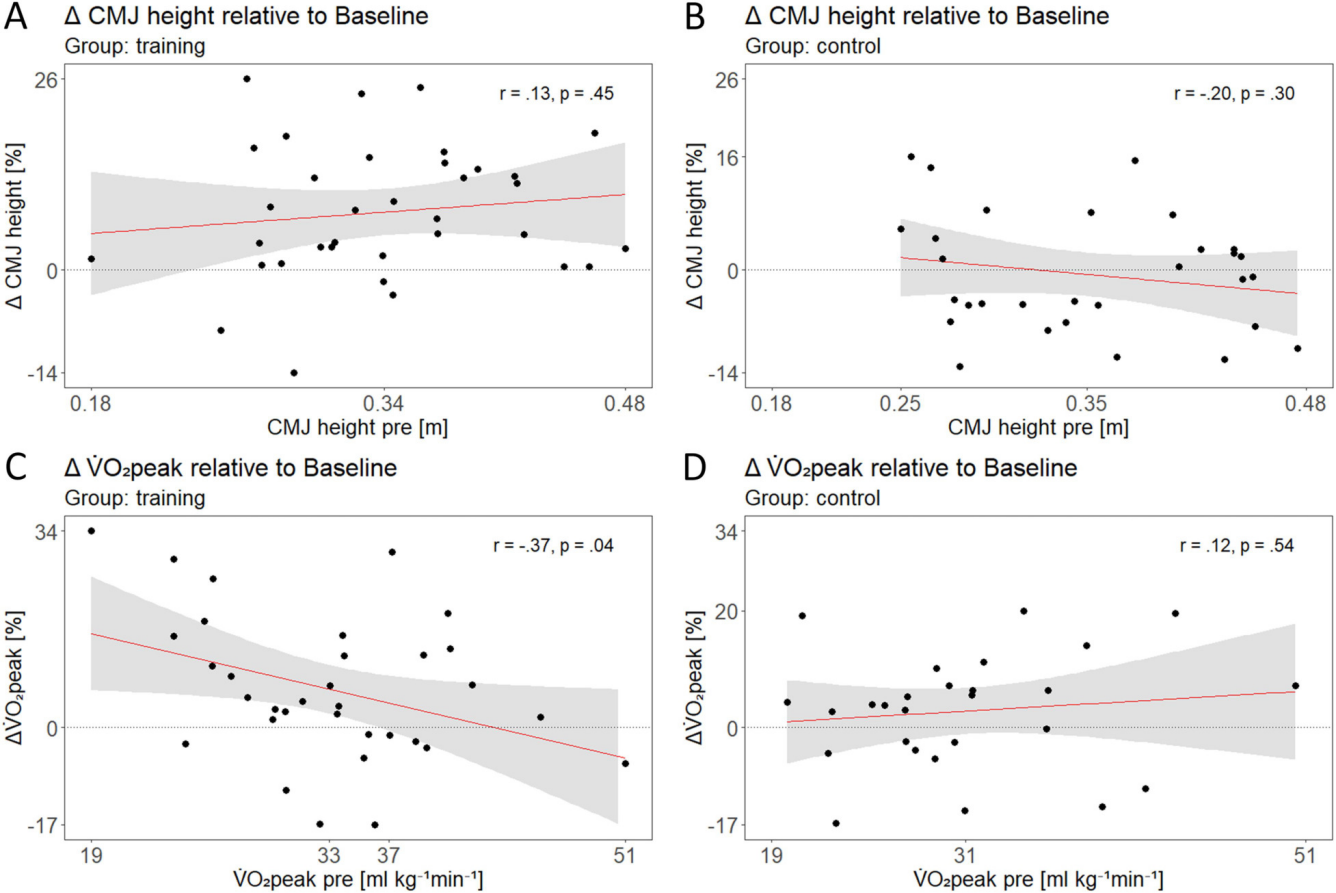
Pre to post + 0 differences in countermovement jump (CMJ) height for the training **(A)** and control **(B)** groups as well as Pre to post +  + 0 $\dot{V}O_{2\mathrm{peak}}$ differences for both groups **(C,D)** are displayed. The solid lines represent fitted linear regression models, with shaded bands indicating 95% confidence intervals. *Y*-axis ticks show 0, as well as the overall minimum and maximum values across groups; group-specific minimum and maximum values are also shown if they deviate by more than 5%. *X*-axis ticks represent the overall minimum and maximum values across groups and, similarly, group-specific minimum and maximum values if they deviate by more than 5%. Mean values are additionally indicated on the *x*-axis, with an additional *x*-axis tick in C (at 37) indicating the lower CI bound equaling 0.

There were no main effects for group nor time but significant group × time interaction effects for all analyzed variables of the CMJ (Table 1). *Post-hoc* analysis showed an increase in jump height (8 ± 9%), higher absolute (4 ± 8%) and relative peak power (5 ± 7%) as well as a greater upward movement range (16 ± 17%) and upward movement and braking duration (19 ± 21%; 31 ± 36%) from Pre to Post + 0 for the training group, while absolute (−8 ± 12%) and relative forces at 0 velocity (−8 ± 11%) were decreased. All changes were maintained at Post + 63, except absolute and relative peak power, which did not significantly differ at Post + 63 compared to Pre or Post + 0. We did not find any changes in the control group for none of the analyzed variables. We then analyzed correlations between changes of jump height from Pre to Post + 0 and jump height at Pre for the training (Figure 2A) and the control group (Figure 2B). Increases in jump height after training were not correlated with baseline levels of jump height in the training group (Figure 2A).

**Table 1:** Overview on the type 3 ANOVA for mixed models results for all performance variables.

Measurement	Variable	Group	Pre	Post + 0	Post + 63	Group	Time	Group × Time	*Post hoc* tests		
									Pre to Post + 0	Pre to Post + 63	Post + 0 to Post + 63
Counter movement jump test	Jump height [m]	*t*	0.34 ± 0.07	0.37 ± 0.08	0.36 ± 0.08	*χ*^2^(1) = 0.45, *p* = 0.501, *η*^2^ = 0	*χ*^2^(2) = 1.42, *p* = 0.492, *η*^2^ = 0.11	*χ*^2^(2) = 27.68, *p* = 0.000***, *η*^2^ = 0.18	*p* = 0.000***, *d* = −1.53	*p* = 0.000***, *d* = −1.19	
		*c*	0.35 ± 0.07	0.35 ± 0.07	0.35 ± 0.06						
	Peak power absolute [kW]	*t*	2.73 ± 0.74	2.84 ± 0.77	2.80 ± 0.76	*χ*^2^(1) = 1.32, *p* = 0.251, *η*^2^ = 0.01	*χ*^2^(2) = 0.88, *p* = 0.643, *η*^2^ = 0.03	*χ*^2^(2) = 9.21, *p* = 0.010*, *η*^2^ = 0.07	*p* = 0.002**, *d* = −0.85		
		*c*	2.97 ± 0.95	2.94 ± 0.95	2.93 ± 0.84						
	Peak power normalized [W kg^−1^]	*t*	39.0 ± 7.1	40.8 ± 8.0	40.1 ± 7.7	*χ*^2^(1) = 0.54, *p* = 0.462, *η*^2^ = 0	*χ*^2^(2) = 1.99, *p* = 0.370, *η*^2^ = 0.03	*χ*^2^(2) = 15.74, *p* = 0.000***, *η*^2^ = 0.11	*p* = 0.000***, *d* = −1.04		
		*c*	40.3 ± 7.3	39.7 ± 7.0	39.9 ± 6.0						
	Force at 0 velocity absolute [kN]	*t*	1.39 ± 0.25	1.27 ± 0.25	1.30 ± 0.25	*χ*^2^(1) = 0.7, *p* = 0.402, *η*^2^ = 0.05	*χ*^2^(2) = 2.42, *p* = 0.298, *η*^2^ = 0.14	*χ*^2^(2) = 9.5, *p* = 0.009**, *η*^2^ = 0.07	*p* = 0.000***, *d* = 1.2	*p* = 0.000***, *d* = 0.96	
		*c*	1.45 ± 0.32	1.42 ± 0.33	1.46 ± 0.34						
	Force at 0 velocity normalized [N kg^−1^]	*t*	20.0 ± 2.2	18.4 ± 2.4	18.7 ± 2.2	*χ*^2^(1) = 0, *p* = 0.998, *η*^2^ = 0.03	*χ*^2^(2) = 3.16, *p* = 0.206, *η*^2^ = 0.15	*χ*^2^(2) = 8.8, *p* = 0.012*, *η*^2^ = 0.07	*p* = 0.000***, *d* = 1.21	*p* = 0.000***, *d* = 1	
		*c*	20.0 ± 2.6	19.5 ± 2.7	20.0 ± 3.1						
	Upward range [m]	*t*	0.39 ± 0.08	0.45 ± 0.07	0.44 ± 0.07	*χ*^2^(1) = 0.12, *p* = 0.724, *η*^2^ = 0.07	*χ*^2^(2) = 1.5, *p* = 0.472, *η*^2^ = 0.2	*χ*^2^(2) = 19.9, *p* = 0.000***, *η*^2^ = 0.14	*p* = 0.000***, *d* = −1.56	*p* = 0.000***, *d* = −1.43	
		*c*	0.38 ± 0.08	0.39 ± 0.08	0.38 ± 0.08						
	Upward duration [ms]	*t*	292 ± 56	342 ± 54	338 ± 54	*χ*^2^(1) = 0.18, *p* = 0.669, *η*^2^ = 0.09	*χ*^2^(2) = 3.31, *p* = 0.191, *η*^2^ = 0.22	*χ*^2^(2) = 14.15, *p* = 0.001**, *η*^2^ = 0.1	*p* = 0.000***, *d* = −1.52	*p* = 0.000***, *d* = −1.38	
		*c*	286 ± 49	301 ± 66	288 ± 65						
	Braking duration [ms]	*t*	200 ± 78	249 ± 68	245 ± 65	*χ*^2^(1) = 0.01, *p* = 0.941, *η*^2^ = 0.04	*χ*^2^(2) = 1.6, *p* = 0.448, *η*^2^ = 0.15	*χ*^2^(2) = 8.53, *p* = 0.014*, *η*^2^ = 0.07	*p* = 0.000***, *d* = −1.19	*p* = 0.000***, *d* = −1.1	
		*c*	199 ± 69	212 ± 81	200 ± 88						
Hop test	Contact time [ms]	*t*	233 ± 34	228 ± 39	224 ± 34	*χ*^2^(1) = 0.37, *p* = 0.543, *η*^2^ = 0.03	*χ*^2^(2) = 1.24, *p* = 0.538, *η*^2^ = 0.02	*χ*^2^(2) = 1.25, *p* = 0.536, *η*^2^ = 0.01			
		*c*	239 ± 42	243 ± 47	234 ± 36						
	Flight time [ms]	*t*	338 ± 63	346 ± 53	348 ± 69	*χ*^2^(1) = 1.21, *p* = 0.272, *η*^2^ = 0.04	*χ*^2^(2) = 0.05, *p* = 0.974, *η*^2^ = 0.01	*χ*^2^(2) = 0.36, *p* = 0.837, *η*^2^ = 0			
		*c*	320 ± 70	322 ± 65	329 ± 72						
	Reactive strength index	*t*	1.49 ± 0.34	1.56 ± 0.34	1.60 ± 0.43	*χ*^2^(1) = 1.12, *p* = 0.290, *η*^2^ = 0.04	*χ*^2^(2) = 0.43, *p* = 0.809, *η*^2^ = 0.04	*χ*^2^(2) = 1.9, *p* = 0.387, *η*^2^ = 0.02			
		*c*	1.38 ± 0.39	1.39 ± 0.43	1.46 ± 0.45						
	Hop height [m]	*t*	0.15 ± 0.05	0.15 ± 0.04	0.15 ± 0.06	*χ*^2^(1) = 1.07, *p* = 0.301, *η*^2^ = 0.03	*χ*^2^(2) = 0.13, *p* = 0.938, *η*^2^ = 0.01	*χ*^2^(2) = 0.36, *p* = 0.835, *η*^2^ = 0			
		*c*	0.13 ± 0.05	0.13 ± 0.05	0.14 ± 0.06						
	Peak force absolute [kN]	*t*	3.31 ± 0.59	3.30 ± 0.61	3.39 ± 0.60	*χ*^2^(1) = 0.01, *p* = 0.941, *η*^2^ = 0	*χ*^2^(2) = 0.51, *p* = 0.774, *η*^2^ = 0.03	*χ*^2^(2) = 0.61, *p* = 0.736, *η*^2^ = 0.01			
		*c*	3.32 ± 0.80	3.31 ± 0.82	3.37 ± 0.82						
	Peak force normalized [N kg^−1^]	*t*	47.7 ± 6.2	47.8 ± 6.5	49.2 ± 7.5	*χ*^2^(1) = 1.47, *p* = 0.225, *η*^2^ = 0.04	*χ*^2^(2) = 0.4, *p* = 0.820, *η*^2^ = 0.03	*χ*^2^(2) = 1.31, *p* = 0.519, *η*^2^ = 0.01			
		*c*	45.6 ± 6.2	45.4 ± 7.3	46.4 ± 7.1						
Leg press test	MVC absolute [N]	*t*	1771 ± 425	1898 ± 494	1730 ± 382	*χ*^2^(1) = 1.72, *p* = 0.190, *η*^2^ = 0.07	*χ*^2^(2) = 2.58, *p* = 0.275, *η*^2^ = 0.12	*χ*^2^(2) = 0.97, *p* = 0.616, *η*^2^ = 0.02			
		*c*	1976 ± 378	2089 ± 489	2007 ± 266						
	MVC normalized [N kg^−1^]	*t*	25.9 ± 4.6	27.8 ± 5.8	25.8 ± 5.1	*χ*^2^(1) = 0.51, *p* = 0.475, *η*^2^ = 0.02	*χ*^2^(2) = 1.43, *p* = 0.489, *η*^2^ = 0.1	*χ*^2^(2) = 1.3, *p* = 0.522, *η*^2^ = 0.02			
		*c*	27.2 ± 4.5	28.3 ± 5.7	27.0 ± 3.0						
One-leg stance test	CoP area [cm^2^]	*t*	4.00 ± 3.02	3.82 ± 2.72	3.43 ± 1.77	*χ*^2^(1) = 0.24, *p* = 0.621, *η*^2^ = 0.02	*χ*^2^(2) = 1.77, *p* = 0.412, *η*^2^ = 0.02	*χ*^2^(2) = 0.83, *p* = 0.662, *η*^2^ = 0.01			
		*c*	3.73 ± 2.80	3.02 ± 1.38	3.28 ± 1.85						
10 m gait test	Velocity [m s^−1^]	*t*	1.73 ± 0.25	1.60 ± 0.24	1.65 ± 0.26	*χ*^2^(1) = 1, *p* = 0.317, *η*^2^ = 0.01	*χ*^2^(2) = 5.19, *p* = 0.074, *η*^2^ = 0.12	*χ*^2^(2) = 0.72, *p* = 0.698, *η*^2^ = 0.01			
		*c*	1.67 ± 0.23	1.58 ± 0.23	1.61 ± 0.26						
	Stride length [cm]	*t*	83.5 ± 9.1	80.3 ± 7.7	80.9 ± 8.5	*χ*^2^(1) = 0.28, *p* = 0.595, *η*^2^ = 0.01	*χ*^2^(2) = 14.76, *p* = 0.001**, *η*^2^ = 0.16	*χ*^2^(2) = 0.81, *p* = 0.668, *η*^2^ = 0.01			
		*c*	82.4 ± 8.7	78.5 ± 7.6	78.6 ± 8.4						
Stair climb test	Time [s]	*t*	8.17 ± 0.84	8.14 ± 1.00	8.00 ± 0.90	*χ*^2^(1) = 1.37, *p* = 0.242, *η*^2^ = 0	*χ*^2^(2) = 17.25, *p* = 0.000***, *η*^2^ = 0.11	*χ*^2^(2) = 5.76, *p* = 0.056, *η*^2^ = 0.04			
		*c*	8.42 ± 0.90	7.95 ± 0.67	7.77 ± 0.76						
	Heart rate increase [%]	*t*	18.4 ± 9.2	24.2 ± 13.3	21.3 ± 11.0	*χ*^2^(1) = 0.01, *p* = 0.941, *η*^2^ = 0	*χ*^2^(2) = 5.77, *p* = 0.056, *η*^2^ = 0.08	*χ*^2^(2) = 0.66, *p* = 0.717, *η*^2^ = 0.01			
		*c*	18.6 ± 13.2	24.6 ± 14.4	18.8 ± 8.8						
Cardiopulmonary exercise test	$\dot{V}O_{2}$ peak [ml kg^−1^ min^−1^]	*t*	33.4 ± 6.8	35.3 ± 7.1	35.6 ± 6.6	*χ*^2^(1) = 1.83, *p* = 0.176, *η*^2^ = 0.05	*χ*^2^(2) = 2.4, *p* = 0.302, *η*^2^ = 0.11	*χ*^2^(2) = 1.82, *p* = 0.403, *η*^2^ = 0.02			
		*c*	30.8 ± 7.2	31.7 ± 8.6	32.0 ± 9.3						
	Max. load absolute [W]	*t*	213 ± 50	222 ± 49	224 ± 52	*χ*^2^(1) = 1.27, *p* = 0.260, *η*^2^ = 0.02	*χ*^2^(2) = 11.47, *p* = 0.003**, *η*^2^ = 0.18	*χ*^2^(2) = 0.52, *p* = 0.773, *η*^2^ = 0			
		*c*	199 ± 46	205 ± 49	213 ± 57						
	Max. load normalized [W kg^−1^]	*t*	3.07 ± 0.62	3.22 ± 0.59	3.24 ± 0.58	*χ*^2^(1) = 2.91, *p* = 0.088, *η*^2^ = 0.06	*χ*^2^(2) = 8.65, *p* = 0.013*, *η*^2^ = 0.17	*χ*^2^(2) = 1, *p* = 0.607, *η*^2^ = 0.01			
		*c*	2.79 ± 0.67	2.88 ± 0.74	2.98 ± 0.80						

The first column subdivides the table into the different tests with the independent variables listed in the second column. Descriptive values are presented as Mean ± SD for the training (*t*) and control (*c*) groups and for Pre, Post + 0 and Post + 63, respectively. ANOVA results are reported using Chi-square (*χ*^2^), *p*-value, and Eta-square (*η*^2^). *Post-hoc* analyses were performed only for variables that demonstrated statistically significant group × time interaction effects and are presented with *p*-values and Cohen's *d*. Significance levels are indicated as follows: *p* < 0.05*, *p* < 0.01**, *p* < 0.001***. Abbreviations: CoP, center of pressure.

Functional principal component analysis on CMJ relative force-time traces revealed that the scores of the first and third component significantly decreased and increased, respectively, from Pre to Post + 0 in the training group only (Supplementary Material S5). These changes indicated reduced relative force throughout the movement after training, except at higher velocities near takeoff, where relative force increased.

**Figure 3:**
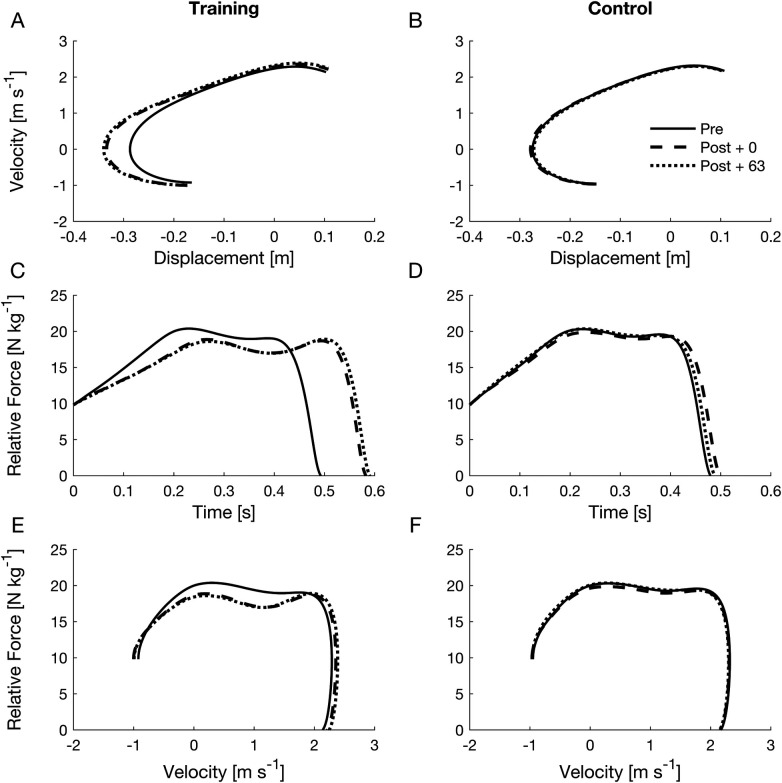
Mean displacement-velocity traces **(A,B)**, relative force-time **(C,D)** and relative force-velocity **(E,F)** for the training and the control group respectively. Displayed traces are group-averaged and normalized; individual variability is not shown. Continuous, dashed, and dotted lines represent the results obtained during Pre, Post + 0, and Post + 63 testing. Only the braking (from peak negative velocity to 0) and upward (from 0 velocity to take off) movement phases were considered. For relative force-time curves, all jumps were interpolated to 401 data points. Piecewise linear length normalization aligned the start and end of the braking and upward movement phases. The braking phase duration across all participants and trials represented ∼71% of the upward movement duration. Therefore, the braking and upward movement phases were interpolated with 167 and 234 data points, respectively. Relative force-time traces were then horizontally rescaled to represent the duration of the braking and upward movement phases. There are apparent changes in the training group between Pre (solid line) and Post +0, Post + 63 (dotted and dashed lines). Participants squatted deeper during the jump which increased displacement **(A)** and time **(B)** This strategy led to a greater impulse and ultimately to a higher take off velocity **(E)** No changes can be observed in the control group **(B,D,F)**.

The mean effect of these changes in the kinetic variables of CMJs for the training group, or lack of them for the control group, is shown in Figure 3, where mean velocity-displacement, relative force-time, and relative force-velocity traces are reported for all testing sessions. As shown by the mean velocity-displacement traces, participants increased the depth of the countermovement after training, exhibiting greater negative displacement values. This resulted in a longer duration of braking and upward movement and a greater range of upward movement, as shown by the shift to the right for the start of the relative force-time curve in the Post + 0 and Post + 63 sessions compared to Pre.

Flight time, contact time, reactive strength index, jump height and absolute and normalized peak force during hops remained unchanged over the measurements at Pre, Post + 0 and Post + 63 (see Table 1). Moreover, there were no significant effects of time, group or group × time interaction for absolute nor relative MVC in the leg press test. We found the same result, no interaction effects, for the one-leg stance, gait, and stair climb tests, while a main effect of time was observed for stride length during gait and stair climb time with shorter stride lengths and shorter stair climb times at Post + 0 and Post + 63 vs. Pre (see Table 1).

When we looked at maximal aerobic capacity, we did not observe a group × time interaction effect for $\dot{V}O_{2\mathrm{peak}}$ nor one for absolute and normalized maximal load during the incremental CPET (see Table 1). However, we found a significant main effect of time for absolute and relative maximal load, with higher values at Post + 0 and Post + 63 compared to Pre. We also saw a correlation between changes in $\dot{V}O_{2\mathrm{peak}}$ between Pre and Post + 0 and baseline levels of $\dot{V}O_{2\mathrm{peak}}$ at Pre, only in the training group with the confidence interval indicating a potential increase in $\dot{V}O_{2\mathrm{peak}}$ after training for participants with Pre baseline values lower than 37 mL kg^−1^min^−1^ (Figures 2C,D).

## 4 Discussion

The 8-week high-intensity jump training effectively promoted task-specific adaptations that enhanced CMJ performance, despite the relatively low weekly training volume of three 15-min sessions. Participants in the training group learned to initiate the movement with a deeper countermovement, thereby lengthening the acceleration path. This adjustment led to an increased peak power output of the lower limbs, reflecting a functional improvement in explosive performance. However, no additional improvements were observed in strength, balance, or generic functional capacity tests. Interestingly, participants with lower baseline aerobic capacity appeared to benefit from the intervention, suggesting that training-induced changes were influenced by baseline fitness levels. Taken together, the observed improvements in CMJ performance, coupled with a high adherence rate (92%) in previously non-exercising young adults, highlight the potential of this program as a feasible countermeasure, although transfer to more generic outcomes was limited.

### 4.1 Adaptations in CMJ performance

Performance improvements in CMJs can primarily be attributed to an optimized movement strategy. A greater countermovement depth increased the time available for impulse generation, thereby enhancing takeoff velocity and jump height. Notably, these benefits came at the expense of relative force during much of the movement. Relative force-velocity traces demonstrated that, after training, force production was reduced during both braking and upward phases, except near takeoff where higher relative forces at greater velocities facilitated elevated peak power output. This observation aligns with recent findings showing that deeper countermovements compromise force generation at low velocities but enhance it at higher velocities, ultimately leading to improved performance (25). As no external feedback was provided during training, these technique-related adaptations likely emerged spontaneously.

Functional principal component analysis confirmed a decrease in relative force across most of the movement, with higher relative force emerging only near takeoff (first fPC and associated scores). No similar changes were seen in the control group, supporting the conclusion that CMJ performance gains were primarily technique-driven. Importantly, CMJ improvements were independent of baseline performance levels. This can be explained by neural plasticity that usually evolves early during training and leads to task specific increases in neuromuscular performance, independent of the initial performance in novices to the trained task (26).

### 4.2 Lack of transfer to hopping performance

Despite hops being an integral training component, hop test performance did not improve. Previous studies have shown that hop height can improve in both older adults and athletes, yet such gains typically stem from changes in the mechanical properties of the muscle–tendon complex rather than altered motor strategies (27, 28). This may explain why CMJ performance improved while hops did not. In this study, neuroplastic adaptations seemed insufficient to affect hop performance, and changes in muscle–tendon function were likely too small to drive measurable improvements (9, 27, 28).

### 4.3 Limited translation to strength and daily function

Although CMJ peak power improved, no training effects were observed in maximal isometric leg extension strength or functional assessments, including one-leg stance, 14-step stair climb, and 10 m gait. These findings are consistent with previous research showing that jump-specific adaptations do not necessarily generalize to unrelated motor tasks (29). It is also plausible that baseline function in these tests was already high, leaving limited scope for further improvement (30–32).

In gait analysis, both groups exhibited reduced stride length and gait velocity after training. As no group-by-time interaction was detected, these changes likely reflect test-related familiarization effects rather than training-induced adaptations (33). This interpretation is supported by the reduced stair climb times observed in both groups at follow-up assessments.

### 4.4 Aerobic performance outcomes

Training also produced baseline-dependent effects on aerobic performance. Within the training group, participants with low baseline aerobic fitness showed clear improvements, whereas those with moderate or high fitness levels did not. This negative correlation likely reflects the overload principle: adaptations occur only if the physiological system is challenged beyond its habitual load (34, 35). Although aerobic training was not a central focus of the intervention, some sessions included continuous jump bouts of ≥3 min, resembling high-intensity interval protocols (36). Despite the limited volume, this load may have been sufficient to induce cardiovascular adaptations in participants with low initial capacity. High-intensity training is generally more effective than moderate continuous exercise for untrained adults (37, 38), but a dose–response relationship has not yet been established for jump training (39).

Evidence from our previous bed rest studies supports the potential of jump training to preserve aerobic capacity. During 60 days of bed rest, a jump training intervention with higher weekly frequency (5–6 sessions) and similar session length almost fully preserved aerobic performance compared to controls (11, 13, 17). In the present study, the lower frequency (3 sessions/week) and absence of confinement likely reduced the overall cardiovascular load, limiting effects to individuals with lower baseline fitness. Exploratory threshold estimation suggested a baseline aerobic capacity below ∼37 mL kg^−1^min^−1^ may be required for measurable improvements, though this finding should be interpreted cautiously as it was not hypothesis-driven. In addition, because baseline $\dot{V}O_{2\mathrm{peak}}$ is included in both the predictor and the relative change score, the observed association may in part be driven by regression-to-the-mean effects.

### 4.5 Practical implications and future directions

The current training program was designed to be scalable, requiring only 15 min per session and three sessions per week. This minimal dose was sufficient to elicit meaningful adaptations in unfit but otherwise healthy young adults compared to baseline levels in literature (40–44). Since it has already been shown that jump training can also elicit more generic improvements in untrained participants (45), future research should evaluate strategies to optimize its effectiveness. Extending session duration (20–30 min), increasing frequency (4–5 sessions per week), or a combination of both may amplify adaptations. Additionally, tailoring training to baseline performance (e.g., focusing more on aerobic vs. anaerobic capacity) may further expand outcomes across diverse populations.

### 4.6 Sample characteristics and generalizability

Participants were recruited as non-exercising young adults. Although the recruitment strategy aimed to identify individuals with relatively low levels of self-reported physical activity, baseline screening data indicated that average MVPA was below but close to the World Health Organization recommendation of at least 150 min/week (46). Thus, as objective activity thresholds were not used as inclusion criteria, the sample can be characterized as non-exercising but relatively active in daily life rather than strictly inactive. This may have limited the potential for training-induced improvements in generic performance outcomes due to ceiling effects. Furthermore, the sample consisted of young, healthy adults, and the present findings may not generalize to older adults, clinical populations, or individuals with mobility limitations. Future studies should therefore incorporate objective activity-based screening procedures to more precisely target low-activity populations and examine the effects of low-volume, high-intensity jump training across older and clinical cohorts. Additionally, continuous activity tracking during the intervention could be useful to better control for potential influences from changes in habitual physical activity.

### 4.7 Conclusion

The 8-week high-intensity jump training proved to be a feasible intervention for a previously non-exercising population, resulting in meaningful improvements in CMJ height and peak power. However, these adaptations did not translate to gains in maximal isometric strength or functional performance measures such as balance, walking, or stair climbing. Aerobic benefits were observed in individuals with low baseline fitness, while overall effects on aerobic performance remained limited. Collectively, these results emphasize the importance of adjusting the load and progression of jump training to the individual capacities of the participants to achieve general enhancements in physical performance.

## Data Availability

The raw data supporting the conclusions of this article will be made available by the authors, without undue reservation.
