# Supplementary material for: Effects of jump training on power, strength, balance and aerobic performance in non-exercising young adults
Source: Front Sports Act Living. 2026 Feb 26;8:1746624. doi: 10.3389/fspor.2026.1746624 (PMC12979136; doi:10.3389/fspor.2026.1746624)
Supplement: Supplementary file 1 [file Datasheet1.pdf]

Supplementary Material S1 - Countermovement jump analysis

Recordings from the two individual force plates were summed up to give an overall force-time curve. The first point deviating from baseline more than 10 standard deviations was determined. The start time of the CMJ was set 0.5 s earlier. Body weight was determined as the average of the vertical force across a 0.5-s second window before start time. CMJ takeoff was automatically identified. The start and takeoff identification for each jump were visually inspected, and if necessary (2 out of 549 jumps) modified. Net force (absolute force minus body weight) as well as the acceleration (net force divided by body mass), velocity and displacement (by integration over time) of the body's center of mass were calculated between the start time and the moment of take-off. Based on the velocity data, the jumping motion was divided into a braking phase (from peak negative velocity to 0) and an upward movement phase (from 0 velocity to take off). The net force was integrated from the end of the braking phase to the end of the propulsive phase to obtain the net propulsive impulse (Ns) and divided by body mass to calculate take-off velocity (ms^{-1}); the airborne displacement (m) was then calculated, and jump height was obtained by summing the airborne displacement and the displacement at take-off. Absolute peak power (absolute force multiplied by velocity, W) and force at the end of the braking phase (force at 0 velocity, kN) were calculated and divided by body mass to obtain relative values ($W\ kg^{-1}$ and $N\ kg^{-1}$). The displacement of the upward movement phase, and the duration of the braking and upward movement phases were then determined.

For comparability with previous literature using field-based methods, jump height was also calculated using flight time in both groups. Regardless of group, in the Pre session, jump height calculated with the flight time method was 0.30 ± 0.04 m for males, and 0.20 ± 0.04 m for females. Results for the training and control group at Pre, Post + 0 and Post + 63 are reported in Supplementary Table 1 below.

Supplementary Table 1. Mean ± SD values of flight time and jump height for the training and control groups across the three testing sessions.

Measurement	Variable	Group	Pre	Post + 0	Post + 63
Counter Movement Jump Test	Flight time [ms]	t	444 ± 63	466 ± 72	460 ± 71
		c	451 ± 60	446 ± 58	443 ± 54
	Jump height [m]	t	0.25 ± 0.07	0.27 ± 0.08	0.27 ± 0.08
		c	0.25 ± 0.06	0.25 ± 0.06	0.24 ± 0.06
