# Supplementary material for: Effects of jump training on power, strength, balance and aerobic performance in non-exercising young adults
Source: Front Sports Act Living. 2026 Feb 26;8:1746624. doi: 10.3389/fspor.2026.1746624 (PMC12979136; doi:10.3389/fspor.2026.1746624)
Supplement: Supplementary file 2 [file Datasheet2.pdf]

Supplementary Material S2 - Hop analysis

Recordings from the 2 individual force plates were summed up to give an overall force-time curve. The peak force of each hop was automatically identified and only hops 4 to 8 were retained for further analysis. Takeoff and landing were determined as the first point below and above 10 N, with a negative and positive first derivative of force, respectively. Contact and flight times [s] were then calculated. The reactive strength index was determined as the ratio between flight time and contact time. Airborne displacement was calculated from flight time. Absolute [kN] and relative (to body mass) [N kg^{-1}] peak force was calculated. Each variable represented the average of the 5 considered hops for each trial.
