# Supplementary material for: Effects of jump training on power, strength, balance and aerobic performance in non-exercising young adults
Source: Front Sports Act Living. 2026 Feb 26;8:1746624. doi: 10.3389/fspor.2026.1746624 (PMC12979136; doi:10.3389/fspor.2026.1746624)
Supplement: Supplementary file 3 [file Datasheet3.pdf]

Supplementary Material S3 - Training programming overview

Supplementary Table 2. Overview of all programming variables per training session. Maximal repetitive CMJs referring to the high-intensity part of the training. Each column represents one training session. Warm-up parts were consistent and are shaded in grey. See Supplementary Material S4 for descriptions and video links of exercises.

Exercise		Week 1			Week 2			Week 3			Week 4			Week 5			Week 6			Week 7			Week 8		
		Training			Training			Training			Training			Training			Training			Training			Training		
		1	2	3	1	2	3	1	2	3	1	2	3	1	2	3	1	2	3	1	2	3	1	2	3
Jumping Jacks	Instructed intensity	moderate																							
	Duration [s]	30																							
	Nr. of repetitions	1																							
	Break after repetition [s]	30																							
	Nr. of sets	1																							
	Break between sets [s]	-																							
Repetitive CMJs	Instructed intensity	moderate																							
	Duration [s]	30																							
	Nr. of repetitions	1																							
	Break after repetition [s]	30																							
	Nr. of sets	1																							
	Break between sets [s]	-																							
High-Knee Runs	Instructed intensity	moderate																							
	Duration [s]	30																							
	Nr. of repetitions	1																							
	Break after repetition [s]	30																							
	Nr. of sets	1																							
	Break between sets [s]	-																							
Hops	Instructed intensity	maximal																							
	Duration [s]	15	15	15	15	15	15	15	15	15	15	15	15	15	15	15	15	15	15	15	15	15	15	15	15
	Nr. of repetitions	6	6	4	4	6	6	6	9	7	7	7	4	4	4	4	4	4	4	4	4	4	4	4	4
	Break after repetition [s]	45	45	45	45	30	30	30	15	15	15	15	15	15	15	15	15	15	15	15	15	15	45	45	45
	Nr. of sets	1	1	1	1	1	1	1	1	1	1	1	1	1	1	1	1	1	1	1	1	1	1	1	1
	Break between sets [s]	-	-	-	-	-	-	-	-	-	-	-	-	-	-	-	-	-	-	-	-	-	-	-	-
Single CMJs	Instructed intensity	maximal																							
	Duration [s]	-	-	-	-	-	-	-	-	-	-	-	-	-	-	-	-	-	-	-	-	-	-	-	-
	Nr. of repetitions	8	8	8	9	9	8	8	8	8	10	10	10	12	12	6	6	6	6	6	-	6	8	8	8
	Break after repetition [s]	40	40	40	30	30	30	25	25	25	20	20	15	15	15	15	15	15	15	15	-	15	40	40	40
	Nr. of sets	1	1	1	1	1	1	1	1	1	1	1	1	1	1	1	1	1	1	1	-	1	1	1	1
	Break between sets [s]	-	-	-	-	-	-	-	-	-	-	-	-	-	-	-	-	-	-	-	-	-	-	-	-
Repetitive CMJs	Instructed intensity	maximal																							
	Duration [s]	30	30	30	30	30	30	30	30	30	30	30	30	30	30	30	30	30	30	30	30	30	30	30	30
	Nr. of repetitions	1	1	3	3	3	4	4	4	5	5	5	3	3	3	4	4	4	4	4	5	4	3	3	3
	Break after repetition [s]	-	-	30	30	30	30	30	30	30	30	30	30	30	30	30	30	30	30	30	30	30	30	30	30
	Nr. of sets	1	1	1	1	1	1	1	1	1	1	1	2	2	2	2	2	2	2	2	2	2	1	1	1
	Break between sets [s]	-	-	-	-	-	-	-	-	-	-	-	60	60	60	60	60	60	60	60	60	60	-	-	-
