# Supplementary material for: Effects of jump training on power, strength, balance and aerobic performance in non-exercising young adults
Source: Front Sports Act Living. 2026 Feb 26;8:1746624. doi: 10.3389/fspor.2026.1746624 (PMC12979136; doi:10.3389/fspor.2026.1746624)
Supplement: Supplementary file 4 [file Datasheet4.pdf]

Supplementary Material S4 - Participants training schedule and training plan

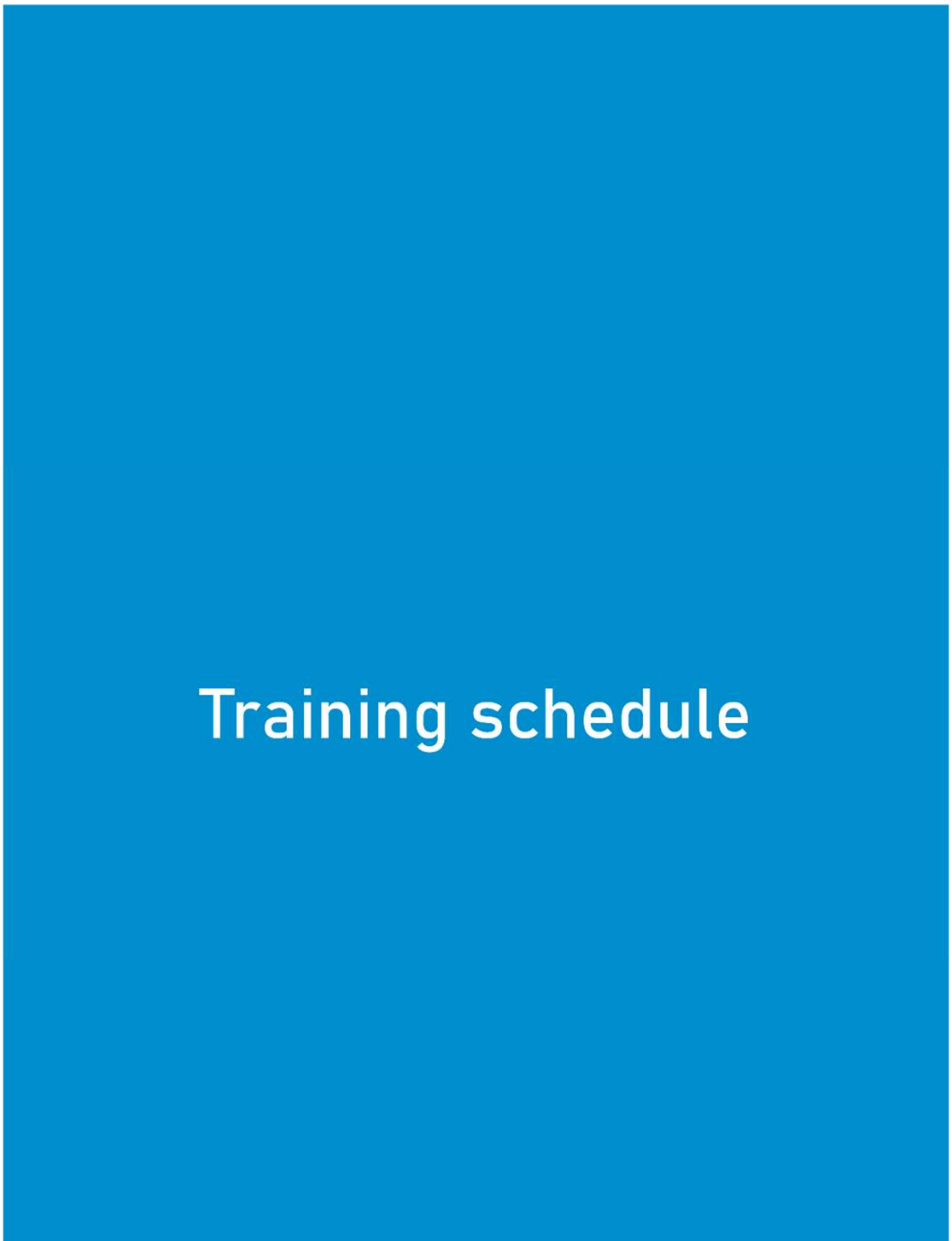

Training schedule

On the following pages, you will find your personal training plan for the next eight weeks. A training session always consists of four components, which are explained below. The repetition and break times vary over the weeks. You will complete three training sessions per week. You should always take at least one day's break between training sessions. Remember to always record your training with your watch and chest strap.

Warm Up	The warm-up is important to prepare your body for exercise. The jumping jacks get your circulation going and activate your leg muscles. The relaxed counter-movement jumps help you prepare your coordination for the jumps during training. The high-knee runs really get you going again. Watch a video of the exercises. →
Hops	When hopping, you jump repetitively on the balls of your feet, similar to jumping rope. Imagine the floor is a hot hob. Watch the video again to see the correct technique. →
CMJs	The aim of the counter-movement jumps during the training session is to jump as high as possible after a short lunge. Maybe you'll manage to touch the ceiling with your head. Watch the video again to see the correct technique. →
HIT	The high-intensity final part of the workout is all about really pushing yourself to the limit. To do this, you repeatedly jump as high as possible in the air. Take another look at the correct technique in the video. →

A training could look like this, for example

No. of training

4

Training progress

		Break time
Warm Up	30s Jumping Jacks<br>30s Counter Movement Jumps<br>30s High-Knee Runs	30s Break
Hops	4x15s Hops	45s Break
CMJs	9x Max. Counter Movement Jump	30s Break
HIT	3x30s repetitive jumping	30s Break

Duration of each part

Checkbox for after the training

Check

Week 1

1

Warm Up	30s Jumping Jacks<br>30s Counter Movement Jumps<br>30s High-Knee Runs	30s Break	
Hops	6x15s Hops	45s Break	
CMJs	8x Max. Counter Movement Jump	40s Break	
HIT	1x30s repetitive jumping	-	Check

2

Warm Up	30s Jumping Jacks<br>30s Counter Movement Jumps<br>30s High-Knee Runs	30s Break	
Hops	6x15s Hops	45s Break	
CMJs	8x Max. Counter Movement Jump	40s Break	
HIT	1x30s repetitive jumping	-	Check

3

Warm Up	30s Jumping Jacks<br>30s Counter Movement Jumps<br>30s High-Knee Runs	30s Break	
Hops	4x15s Hops	45s Break	
CMJs	8x Max. Counter Movement Jump	40s Break	
HIT	3x30s repetitive jumping	30s Break	Check

Week 2

4

Warm Up	30s Jumping Jacks<br>30s Counter Movement Jumps<br>30s High-Knee Runs	30s Break	
Hops	4x15s Hops	45s Break	
CMJs	9x Max. Counter Movement Jump	30s Break	
HIT	3x30s repetitive jumping	30s Break	Check

5

Warm Up	30s Jumping Jacks<br>30s Counter Movement Jumps<br>30s High-Knee Runs	30s Break	
Hops	6x15s Hops	30s Break	
CMJs	9x Max. Counter Movement Jump	30s Break	
HIT	3x30s repetitive jumping	30s Break	Check

6

Warm Up	30s Jumping Jacks<br>30s Counter Movement Jumps<br>30s High-Knee Runs	30s Break	
Hops	6x15s Hops	30s Break	
CMJs	8x Max. Counter Movement Jump	30s Break	
HIT	4x30s repetitive jumping	30s Break	Check

Week 3

7

Warm Up	30s Jumping Jacks<br>30s Counter Movement Jumps<br>30s High-Knee Runs	30s Break	
Hops	6x15s Hops	30s Break	
CMJs	8x Max. Counter Movement Jump	25s Break	
HIT	4x30s repetitive jumping	30s Break	Check

8

Warm Up	30s Jumping Jacks<br>30s Counter Movement Jumps<br>30s High-Knee Runs	30s Break	
Hops	9x15s Hops	15s Break	
CMJs	8x Max. Counter Movement Jump	25s Break	
HIT	4x30s repetitive jumping	30s Break	Check

9

Warm Up	30s Jumping Jacks<br>30s Counter Movement Jumps<br>30s High-Knee Runs	30s Break	
Hops	7x15s Hops	15s Break	
CMJs	8x Max. Counter Movement Jump	25s Break	
HIT	5x30s repetitive jumping	30s Break	Check

Week 4

10

Warm Up	30s Jumping Jacks<br>30s Counter Movement Jumps<br>30s High-Knee Runs	30s Break	
Hops	7x15s Hops	15s Break	
CMJs	10x Max. Counter Movement Jump	20s Break	
HIT	5x30s repetitive jumping	30s Break	Check

11

Warm Up	30s Jumping Jacks<br>30s Counter Movement Jumps<br>30s High-Knee Runs	30s Break	
Hops	7x15s Hops	15s Break	
CMJs	10x Max. Counter Movement Jump	20s Break	
HIT	5x30s repetitive jumping	30s Break	Check

12

Warm Up	30s Jumping Jacks<br>30s Counter Movement Jumps<br>30s High-Knee Runs	30s Break	
Hops	4x15s Hops	15s Break	
CMJs	10x Max. Counter Movement Jump	15s Break	
HIT	3x30s repetitive jumping<br>1min Break<br>3x30s repetitive jumping	30s Break<br>30s Break	Check

Week 5

13

Warm Up	30s Jumping Jacks<br>30s Counter Movement Jumps<br>30s High-Knee Runs	30s Break
Hops	4x15s Hops	15s Break
CMJs	12x Max. Counter Movement Jump	15s Break
HIT	3x30s repetitive jumping<br>1min Break<br>3x30s repetitive jumping	30s Break<br>30s Break

Check

14

Warm Up	30s Jumping Jacks<br>30s Counter Movement Jumps<br>30s High-Knee Runs	30s Break
Hops	4x15s Hops	15s Break
CMJs	12x Max. Counter Movement Jump	15s Break
HIT	3x30s repetitive jumping<br>1min Break<br>3x30s repetitive jumping	30s Break<br>30s Break

Check

15

Warm Up	30s Jumping Jacks<br>30s Counter Movement Jumps<br>30s High-Knee Runs	30s Break
Hops	4x15s Hops	15s Break
CMJs	6x Max. Counter Movement Jump	15s Break
HIT	4x30s repetitive jumping<br>1min Break<br>4x30s repetitive jumping	30s Break<br>30s Break

Check

Week 6

16

Warm Up	30s Jumping Jacks<br>30s Counter Movement Jumps<br>30s High-Knee Runs	30s Break
Hops	4x15s Hops	15s Break
CMJs	6x Max. Counter Movement Jump	15s Break
HIT	4x30s repetitive jumping<br>1min Break<br>4x30s repetitive jumping	30s Break<br>30s Break

Check

17

Warm Up	30s Jumping Jacks<br>30s Counter Movement Jumps<br>30s High-Knee Runs	30s Break
Hops	4x15s Hops	15s Break
CMJs	6x Max. Counter Movement Jump	15s Break
HIT	4x30s repetitive jumping<br>1min Break<br>4x30s repetitive jumping	30s Break<br>30s Break

Check

18

Warm Up	30s Jumping Jacks<br>30s Counter Movement Jumps<br>30s High-Knee Runs	30s Break
Hops	4x15s Hops	15s Break
CMJs	6x Max. Counter Movement Jump	15s Break
HIT	4x30s repetitive jumping<br>1min Break<br>4x30s repetitive jumping	30s Break<br>30s Break

Check

Week 7

19

Warm Up	30s Jumping Jacks<br>30s Counter Movement Jumps<br>30s High-Knee Runs	30s Break	
Hops	4x15s Hops	15s Break	
CMJs	6x Max. Counter Movement Jump	15s Break	
HIT	4x30s repetitive jumping<br>1min Break<br>4x30s repetitive jumping	30s Break<br>30s Break	Check

20

Warm Up	30s Jumping Jacks<br>30s Counter Movement Jumps<br>30s High-Knee Runs	30s Break	
Hops	4x15s Hops	15s Break	
CMJs	-	-	
HIT	5x30s repetitive jumping<br>1min Break<br>5x30s repetitive jumping	30s Break<br>30s Break	Check

21

Warm Up	30s Jumping Jacks<br>30s Counter Movement Jumps<br>30s High-Knee Runs	30s Break	
Hops	4x15s Hops	15s Break	
CMJs	6x Max. Counter Movement Jump	15s Break	
HIT	4x30s repetitive jumping<br>1min Break<br>4x30s repetitive jumping	30s Break<br>30s Break	Check

Week 8

22

Warm Up	30s Jumping Jacks<br>30s Counter Movement Jumps<br>30s High-Knee Runs	30s Break	
Hops	4x15s Hops	45s Break	
CMJs	8x Max. Counter Movement Jump	40s Break	
HIT	3x30s repetitive jumping	30s Break	Check

23

Warm Up	30s Jumping Jacks<br>30s Counter Movement Jumps<br>30s High-Knee Runs	30s Break	
Hops	4x15s Hops	45s Break	
CMJs	8x Max. Counter Movement Jump	40s Break	
HIT	3x30s repetitive jumping	30s Break	Check

24

Warm Up	30s Jumping Jacks<br>30s Counter Movement Jumps<br>30s High-Knee Runs	30s Break	
Hops	4x15s Hops	45s Break	
CMJs	8x Max. Counter Movement Jump	40s Break	
HIT	3x30s repetitive jumping	30s Break	Check
