# Supplementary material for: Effects of jump training on power, strength, balance and aerobic performance in non-exercising young adults
Source: Front Sports Act Living. 2026 Feb 26;8:1746624. doi: 10.3389/fspor.2026.1746624 (PMC12979136; doi:10.3389/fspor.2026.1746624)
Supplement: Supplementary file 5 [file Datasheet5.pdf]

Supplementary Material S5 - Functional principal component analysis of relative force-time curves of CMJs

For functional principal components analysis, only relative force of the braking and upward movement phases at Pre and at Post + 0 for both groups were considered. All jumps were interpolated to 401 data points. Piecewise linear length normalization aligned the start and end of the braking and upward movement phases. The braking phase duration across all participants and trials represented ~71% of the upward movement duration. Therefore, the braking and upward movement phases were interpolated with 167 and 234 data points, respectively. Each curve was estimated using a B-spline basis system. Using a combination of generalized cross-validation and visual confirmation, a roughness penalty (λ) was added for fitting relative force data. No varimax rotation was applied. The first 4 functional principal components (fPCs) and their corresponding scores were considered, retaining 97.6% of the variance of the data. To improve visual interpretation of the components, fPCs were added and subtracted from the mean function and rescaled to absolute units. Supplementary Figure 1 represents interpolated mean relative force–time traces with the fPCs, as well as changes between Pre and Post + 0 in fPC scores in both the training and control groups.

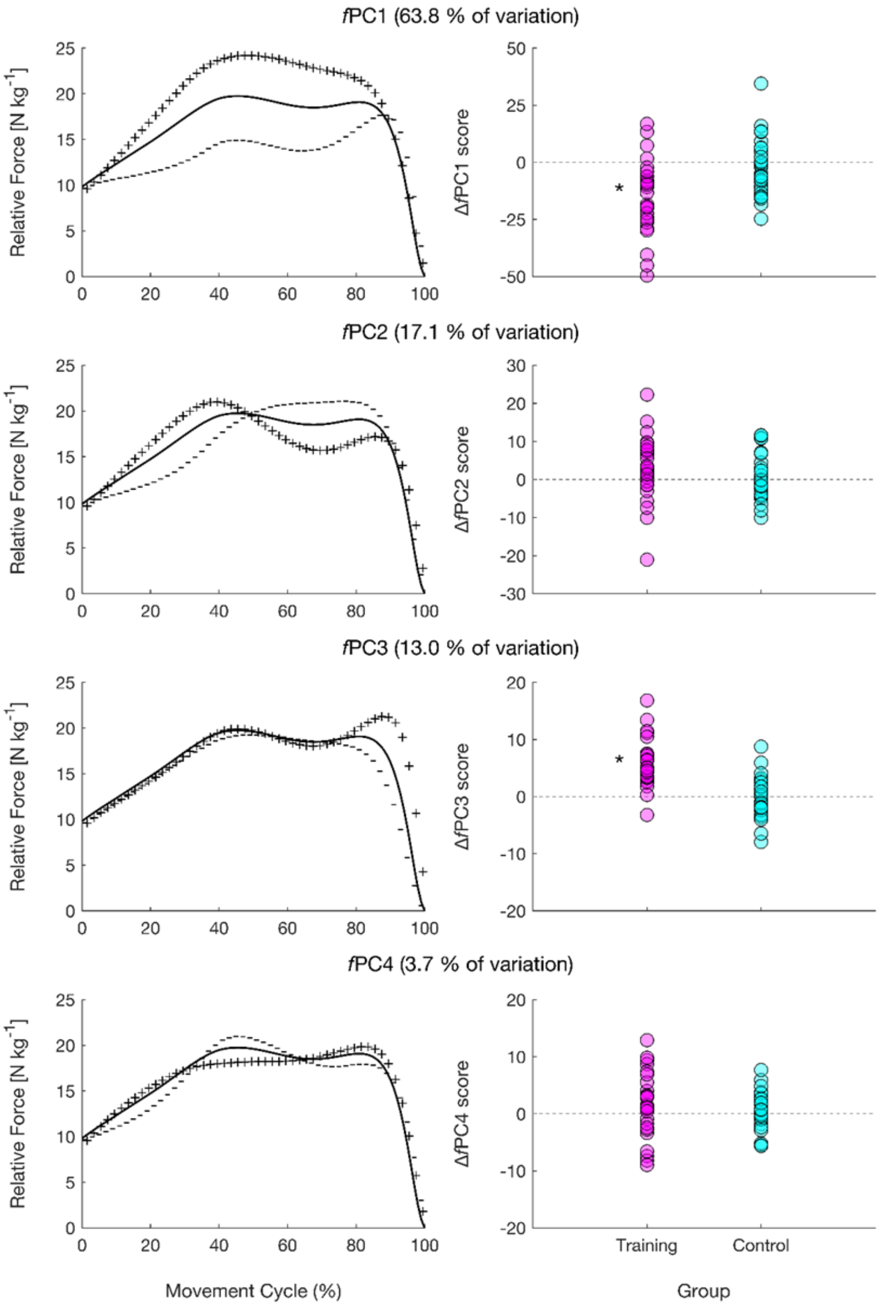

Supplementary Figure 1. Functional principal component analysis of the relative force–time curves of the countermovement jumps at Pre and at Post + 0 in both training and control groups. The first 4 fPCs, explaining 97.6% of the variance of the data, and the associated individual functional principal component (fPC) scores were retained. The curves with + and − in plots show the combined interaction of relative force and time for positive and negative fPC scorers, respectively. In the right column, plots indicate changes in fPC scores in the training (magenta) and control (cyan) groups from Pre to Post + 0. Only for the training group, the scores of fPC1 and 3 decreased and increased (t-test; $*p < 0.05$), respectively, indicating that in the training group the first peak of relative force was lower, while the second peak shifted to the right.
