# Supplementary material for: Effects of jump training on power, strength, balance and aerobic performance in non-exercising young adults
Source: Front Sports Act Living. 2026 Feb 26;8:1746624. doi: 10.3389/fspor.2026.1746624 (PMC12979136; doi:10.3389/fspor.2026.1746624)
Supplement: Supplementary file 6 [file Datasheet6.pdf]

Supplementary Material S6 – Baseline group comparisons

Supplementary Table 3. Baseline comparisons between the training and control groups prior to the intervention for all reported variables. Group differences were assessed using Welch's independent-samples t-tests. Group means are reported in the manuscript.

Measurement	Variable	*t* (df)	*p*
Participant characteristics	Age [y]	-0.63 (60.9)	0.531
	Body height [cm]	0.43 (60.8)	0.671
	Body mass [kg]	1.05 (51.2)	0.300
	BMI [kg m^{-2}]	0.87 (58.3)	0.390
	Resting heart rate [min^{-1}]	-0.21 (61.8)	0.832
	Systolic blood pressure [mmHg]	-0.30 (61.5)	0.763
	Diastolic blood pressure [mmHg]	-0.90 (63.0)	0.370
	Stage of change in physical activity (1-5)	-0.35 (62.8)	0.725
	Moderate-to-vigorous physical activity [min/week]	-0.12 (53.0)	0.902
	Self-rated physical fitness level (1-5)	-0.85 (59.5)	0.400
	Self-rated physical fitness percentile [%]	-1.22 (58.6)	0.226
Counter Movement Jump Test	Jump height [m]	0.70 (60.4)	0.487
	Peak power absolute [kW]	1.12 (54.3)	0.269
	Peak power normalized [W kg^{-1}]	0.74 (60.9)	0.462
	Force at 0 velocity absolute [kN]	0.83 (54.1)	0.413
	Force at 0 velocity normalized [N kg^{-1}]	0.00 (57.0)	0.997
	Upward range [m]	-0.35 (62.3)	0.727
	Upward duration [ms]	-0.47 (63.0)	0.640
	Braking duration [ms]	-0.08 (62.9)	0.939
Hop Test	Contact time [ms]	0.61 (56.2)	0.544
	Flight time [ms]	-1.08 (58.8)	0.283
	Reactive strength index	-1.14 (58.4)	0.257
	Hop height [m]	-1.04 (60.1)	0.301
	Peak force absolute [kN]	0.07 (52.9)	0.941
	Peak force normalized [N kg^{-1}]	-1.34 (61.5)	0.185
Leg Press Test	MVC absolute [N]	1.43 (25.4)	0.165
	MVC normalized [N kg^{-1}]	0.79 (23.4)	0.435
One-leg Stance Test	CoP area [cm^2]	-0.38 (61.8)	0.708
10m Gait Test	Velocity [m s^{-1}]	-1.03 (62.3)	0.309
	Stride length [cm]	-0.50 (62.1)	0.617
Stair Climb Test	Time [s]	1.15 (60.0)	0.256
	Heart rate increase [%]	0.08 (50.6)	0.939
Cardiopulmonary Exercise Test	$\dot{V}O_2$peak [ml kg^{-1} min^{-1}]	-1.44 (54.3)	0.156
	Max. load absolute [W]	-1.18 (62.7)	0.244
	Max. load normalized [W kg^{-1}]	-1.74 (59.9)	0.086
